# Interfacial Charge-Transfer Engineering in Borophene–MWCNT Heterostructures for Multifunctional Humidity and Physiological Sensing

**DOI:** 10.3390/s26030976

**Published:** 2026-02-02

**Authors:** Anran Ma, Tao Wang, Zhilin Zhao, Yi Liu, Maoping Xu, Shengxiang Gao, Rui Zhu, Jiamin Wu, Chuang Hou, Guoan Tai

**Affiliations:** 1 State Key Laboratory of Mechanics and Control for Aerospace Structures, Laboratory of Intelligent Nano Materials, Devices of Ministry of Education, College of Aerospace Engineering, Nanjing University of Aeronautics and Astronautics, Nanjing 210016, China; 2 China Aerospace Science & Industry Academy of Information Technology, Beijing 100070, China; 3 Research Center for Advanced Science and Technology, The University of Tokyo, Tokyo 153-8505, Japan

**Keywords:** heterostructures, interfacial charge-transfer engineering, chemical vapor deposition, humidity sensor, intelligent healthcare monitoring

## Abstract

Humidity sensing is essential in medical fields such as respiratory support, neonatal care, sterilization, and pharmaceutical storage. However, current sensors face limitations, including slow response/recovery, low sensitivity, and poor long-term stability. To address these challenges, we developed borophene-multiwalled carbon nanotube (MWCNT) heterostructures using a stepwise in situ thermal decomposition method. The resulting humidity sensor exhibits an ultrabroad detection range (11–97% RH), ultra-high sensitivity (55,000% at 97% RH), and fast response/recovery times (10.04 s/4.8 s). Through interfacial charge-transfer engineering, the system facilitates rapid electron migration, enhances Schottky barrier modulation, and provides abundant active adsorption sites for water molecules, thereby achieving comprehensive improvement in sensing performance. It also demonstrates excellent selectivity, mechanical flexibility, and operational stability. Notably, the sensor’s sensitivity at 97% RH surpasses that of sensors based on pure borophene or MWCNT by 37–462 times, highlighting the advantages of heterostructure engineering. The multifunctionality of the device suggests its potential in areas beyond conventional sensing, including non-contact voice recognition, skin humidity mapping, and real-time breath monitoring. These results lay a solid foundation for developing borophene-MWCNT heterostructures into a high-performance platform for next-generation medical diagnostics and intelligent health monitoring.

## 1. Introduction

Borophene, a two-dimensional (2D) allotrope of boron, comprises atomically thin layers with unique planar configurations. While structurally reminiscent of graphene, borophene exhibits fundamentally distinct electronic properties due to its versatile bonding and anisotropic lattice structure. Unlike the isotropic behavior of graphene, borophene demonstrates pronounced in-plane anisotropy, high optical transparency, and even phonon-mediated superconductivity, making it a promising candidate for flexible electronics, superconducting circuits, and quantum information technologies [1,2,3,4,5,6,7]. These exceptional physicochemical properties render borophene a compelling candidate for next-generation flexible electronics, superconducting circuits, and quantum information storage systems [8,9,10,11,12]. Although theoretical predictions of borophene date back to the late 20th century [13,14], its experimental synthesis was not achieved until 2015, when Tai et al. reported the growth of γ-phase borophene on copper substrates via chemical vapor deposition (CVD) [2]. This milestone was followed by the successful fabrication of multiple polymorphs—including χ_3_, χ_6_, β_12_, and δ_5_ phases—on metallic substrates such as silver, gold, iridium, and aluminum using molecular beam epitaxy (MBE), all exhibiting metallic conductivity as verified by spectroscopic analyses [9,15,16,17,18]. A major breakthrough occurred in 2020 with the synthesis of semiconducting α′-4H borophene via in situ thermal decomposition of sodium borohydride (NaBH_4_), enabling more scalable and stable borophene fabrication [12].

Despite these advances, the application of borophene in device-level heterostructures remains in its infancy. Environmental instability and challenging synthesis protocols continue to hinder its practical deployment [19]. Nevertheless, heterostructure engineering presents a powerful route to tailor interfacial band alignment, optimize charge transfer dynamics, and modulate carrier transport pathways-capabilities crucial for high-performance applications in optoelectronics, energy conversion, and sensing [20,21]. Specifically, heterointerfaces can significantly improve sensor responsiveness and selectivity by facilitating interfacial charge transfer and providing abundant active adsorption sites.

Multi-walled carbon nanotube (MWCNT), known for their excellent electrical conductivity, high mechanical strength, and large surface area, have been extensively employed in sensing, field emission, and nanoelectronic devices [22]. Their ability to physically and chemically interact with target analytes makes them highly attractive as sensing materials. However, MWCNT-based sensors often suffer from limited sensitivity and inconsistent response behavior. Integrating MWCNT with borophene to form heterostructures can overcome these shortcomings, enabling synergistic enhancement in electronic coupling, structural stability, and sensing performance.

In this work, we report the rational design and synthesis of borophene-MWCNT heterostructures via a tailored CVD approach. Through an interfacial charge-transfer engineering strategy, semiconducting borophene is coupled with a conductive multi-walled carbon nanotube (MWCNT) framework to establish a new material platform that simultaneously delivers high stability, efficient carrier transport, and multifunctional humidity response. This strategy ensures intimate interfacial contact and strong electronic coupling between the two materials. Structural and chemical analyses using scanning electron microscopy (SEM), transmission electron microscopy (TEM), atomic force microscopy (AFM), X-ray photoelectron spectroscopy (XPS), Raman spectroscopy and ultraviolet photoelectron spectroscopy (UPS) confirm the successful integration and preservation of individual material characteristics. The resulting humidity sensor demonstrates an ultrabroad detection range, ultrahigh sensitivity, rapid response and recovery times, and long-term operational stability under ambient conditions. Notably, the sensor also exhibits exceptional selectivity and versatility, enabling practical functionalities such as non-contact voice recognition, respiratory monitoring, and epidermal humidity mapping. These results highlight the potential of borophene-MWCNT heterostructures for next-generation wearable and intelligent sensing systems, offering new perspectives and a material foundation for exploring their applications in healthcare, human–machine interfaces, and environmental monitoring.

## 2. Materials and Methods

### 2.1. Material Synthesis

Sodium borohydride (NaBH_4_, 98%) and ethanol were procured from Aladdin Reagent Co., Ltd. (Shanghai, China), while multi-walled carbon nanotube (MWCNT, >95%) was obtained from XFNANO Materials Technology Co., Ltd. (Nanjing, China). Ultrapure water (18.25 MΩ·cm at 25 °C) was supplied by a Milli-Q purification system and used throughout all experimental procedures. All reagents were of analytical grade and employed without further purification unless otherwise specified. Borophene-MWCNT heterostructures were synthesized via a previously established stepwise in situ thermal decomposition method [12], optimized for the formation of metal-substrate-free 2D hybrids.

Briefly, NaBH_4_ and MWCNT were uniformly mixed at varying molar ratios of 5:1, 10:1, 30:1, and 50:1 (exact mass: 79.17 mg/5.00 mg; 158.00 mg/5.00 mg; 474.00 mg/5.00 mg; 500.00 mg/3.20 mg), followed by controlled thermal treatment under inert conditions to promote the in situ generation of borophene nanosheets and their direct integration with the carbon nanotube framework. Post-synthesis, the resultant borophene-MWCNT composites were dispersed in ultrapure water and subjected to ultrasonic agitation in an ultrasonic cleaner to ensure uniform dispersion and effective exfoliation. The ultrasonic power was set to 100%, and the sonication time was 10 min. Subsequently, the suspensions were placed in a centrifuge and centrifuged at 10,000 rpm for 10 min to remove unbound residues and larger agglomerates. This dispersion-centrifugation cycle was repeated multiple times to ensure high-purity composite acquisition, effectively eliminating unreacted precursors and byproducts. The resulting hybrids exhibited excellent dispersion stability, laying the groundwork for subsequent device fabrication and performance evaluation.

### 2.2. Material Characterizations

The morphological characterization of the heterostructures was carried out using field-emission scanning electron microscopy (FESEM, Nano-SEM 450, Thermo Fisher Scientific, Waltham, MA, USA). The thickness of borophene, MWCNT, and the heterostructures was measured using atomic force microscopy (AFM, Smart SPM, HORIBA Scientific, Irvine, CA, USA). Transmission electron microscopy (TEM) images and selected-area electron diffraction (SAED) patterns were obtained using an F200X field-emission transmission electron microscope (Thermo Fisher Scientific), operating at an acceleration voltage of 200 kV. Elemental mapping was performed with a high-angle annular dark-field (HAADF) detector (SuperX, Milaca, MN, USA) in scanning transmission electron microscopy (STEM) mode. The bonding structure and surface composition of the heterostructures were analyzed using Horiba Evolutionary Raman Microscopy (HORIBA Scientific, 325 nm He-Ne laser, Kyoto, Japan) and X-ray photoelectron spectroscopy (XPS, 250Xi, Science Company, Littleton, CO, USA). Ultraviolet photoelectron spectroscopy (UPS) was measured to calculate the work functions of borophene and MWCNT, thereby elucidating the interfacial electronic band alignment and charge transfer properties of the heterostructures.

### 2.3. Device Fabrication and Measurements

To fabricate the humidity sensing device, 1 mg of borophene-MWCNT heterostructures composite was uniformly dispersed in 1 mL of ethanol via ultrasonication to form a stable colloidal suspension. Subsequently, the dispersion was drop-cast onto a pre-cleaned quartz substrate (1.5 × 1.5 cm^2^) integrated with pre-patterned interdigitated gold electrodes (electrode length: 5 mm; width: 0.5 mm; thickness: 100 nm). The substrate was then subjected to thermal drying at 65 °C for 2 h to ensure solvent evaporation and strong adhesion of the sensing layer. Electrical characterization of the resulting device was conducted at room temperature using a precision source measurement unit (Keithley 2450) under a constant bias voltage of 0.5 V. Time-resolved current measurements (I-t) were recorded to evaluate the real-time humidity sensing performance. The sensor’s response time and recovery time were defined as the durations required to reach 90% of the maximum signal change upon exposure to and removal from the humid environment, corresponding to adsorption and desorption processes, respectively. These parameters served as key indicators of the sensor’s dynamic behavior and operational efficiency.

## 3. Results and Discussion

### 3.1. Synthesis and Preparation of Borophene-MWCNT

**Figure 1:**
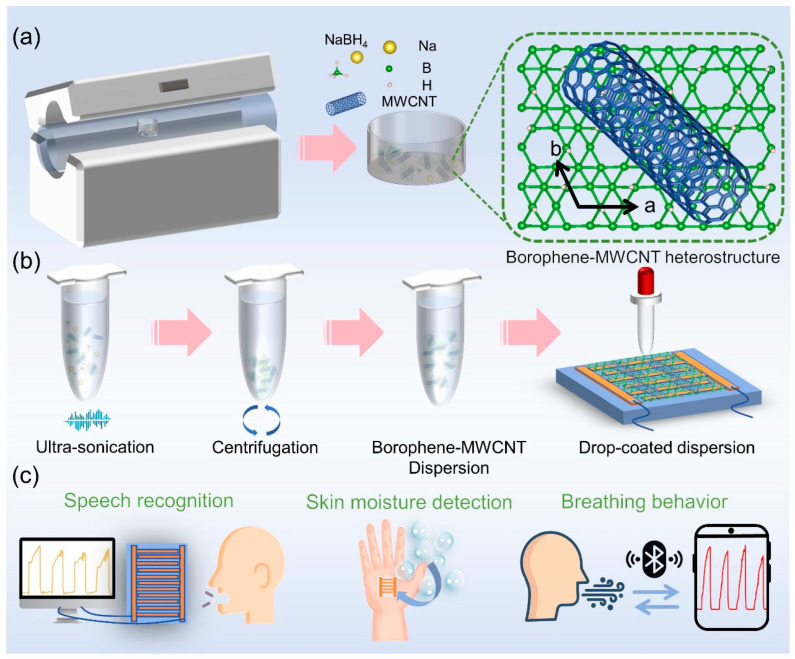
Preparation and Application of Borophene-MWCNT Heterostructures. (**a**) Schematic of the CVD setup used for synthesizing borophene-MWCNT heterostructures. (**b**) Illustration of the cleaning and post-treatment process of the heterostructures. (**c**) Application of the heterostructures in humidity sensing devices.

The borophene-MWCNT heterostructures were synthesized via a modified chemical vapor deposition (CVD) strategy employing a tube furnace system, as schematically illustrated in Figure 1, which outlines both the heterostructures fabrication and sensor assembly processes. Building upon our previously established stepwise in situ thermal decomposition protocol, sodium borohydride (NaBH_4_) and multi-walled carbon nanotube (MWCNT) were combined in predetermined molar ratios and simultaneously subjected to controlled thermal treatment to promote the formation of high-purity, metal-substrate-free borophene-MWCNT heterostructures. The optimized heterostructures, prepared at a borophene-to-MWCNT molar ratio of 10:1, exhibited superior uniformity and dispersion stability. These composites were dispersed in ultrapure water and processed via ultrasonication to ensure homogeneity, followed by centrifugation to remove unreacted precursors and residual impurities. The purified dispersion was then drop-cast onto pre-patterned electrode substrates to fabricate the sensing device. The growth curve is depicted in Figure S1, while comprehensive experimental details are provided in the experimental section.

### 3.2. Comprehensive Characterization of Borophene-MWCNT

**Figure 2:**
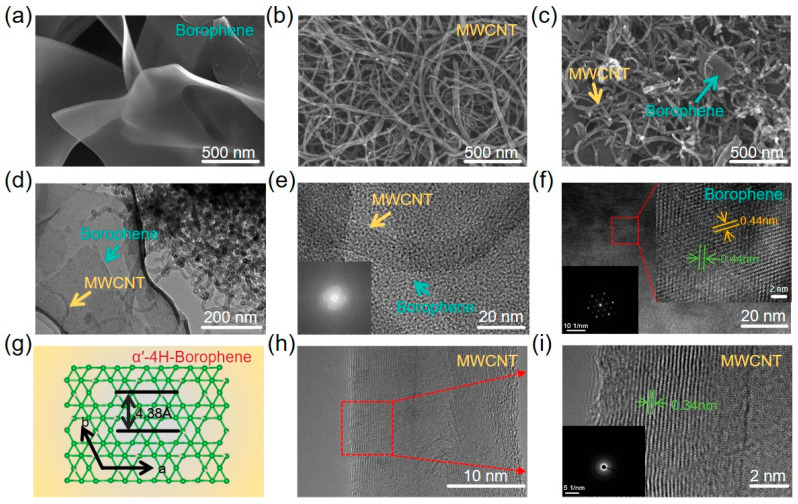
Structural Characterization of Borophene-MWCNT Heterostructures. (**a**–**c**) SEM images of few-layer borophene, MWCNT, and borophene-MWCNT heterostructures, respectively. (**d**) Low-magnification TEM images of the heterostructures (**e**) High-resolution TEM images of the heterostructures. The inset shows the corresponding Fast Fourier Transform (FFT) pattern. (**f**) High-resolution TEM images of borophene. The inset in the lower left shows the SAED pattern of borophene. (**g**) Schematic atomic model of the hydrogenated α’-4H-borophene structure. (**h**,**i**) High-resolution TEM images of MWCNT. The inset in panel (**i**) shows the SAED pattern of MWCNT.

The morphological features of borophene, MWCNT, and their resulting heterostructures were systematically investigated by scanning electron microscopy (SEM), as shown in Figure 2a–c. The pristine borophene nanosheets presented smooth, continuous surfaces with characteristic ultrathin morphology, indicative of their two-dimensional crystalline nature (Figure S2a,b). In contrast, the MWCNT exhibited well-defined, high-aspect-ratio tubular structures with typical lengths of ~50 μm and diameters of 11 nm (Figure S2c,d), consistent with their known nanostructural attributes. The borophene-MWCNT heterostructures formed by hybridization exhibit a hierarchically integrated architecture, where the two components are interwoven and interconnected to construct a robust composite framework (Figure 2c and Figure S2e,f). This intimate interfacial contact provides preliminary confirmation of the successful construction of the heterostructures. Atomic force microscopy (AFM) analysis was performed to quantitatively characterize the surface morphology and thickness of borophene, MWCNT, and borophene-MWCNT heterostructures. As shown in Figure S3a, borophene exists in the form of nanosheets with a thickness distribution around approximately 4 nm (see inset). Meanwhile, Figure S3b displays a porous network structure formed by entangled MWCNT with a typical height of about 22 nm. Following in situ growth, borophene and MWCNT jointly formed a closely integrated composite morphology (Figure S3c). Under the present processing conditions, no large-scale borophene agglomeration or obvious exposure of bare MWCNT regions was observed, indicating good interfacial coupling and uniform spatial distribution between the two phases.

To gain deeper insights into the nanoscale structural features of the borophene-MWCNT heterostructures, comprehensive transmission electron microscopy (TEM) analyses were carried out. Low-magnification TEM images revealed an intimate interfacial integration between the borophene nanosheets and the MWCNT frameworks, directly confirming the successful assembly of the heterostructures (Figure 2d). Additionally, more detailed high-magnification TEM images provided in the Supporting Information (Figure S4) further corroborate this intimate interfacial contact. To provide clearer structural insights into the interface, high-magnification TEM analysis accompanied by a Fast Fourier Transform (FFT) pattern was performed (Figure 2e). High-resolution TEM (HRTEM) further unveiled the well-defined crystalline structure of borophene, with a measured lattice spacing of approximately 0.44 nm (Figure 2f). Notably, the Selected Area Electron Diffraction (SAED) pattern (Figure 2f, inset) unambiguously confirms the phase purity and high crystallinity, consistent with the characteristic interplanar distance of hydrogenated α′-4H borophene (Figure 2g), as reported in prior literature [12,19]. In parallel, the MWCNTs maintained their canonical multiwalled architecture, exhibiting entangled hollow tubular forms with a typical interlayer spacing of ~0.34 nm (Figure 2h,i), where the structural integrity was further validated by the corresponding SAED pattern (Figure 2i, inset). Collectively, these microscopic characterizations demonstrate the successful physical assembly and close structural contact of the heterostructure components.

To further elucidate the spatial organization and compositional uniformity, high-angle annular dark-field scanning transmission electron microscopy (HAADF-STEM) was performed on representative regions (Figure S5). The HAADF-STEM images reveal a well-integrated composite morphology. Elemental mapping (Figure S5) further confirms the homogeneous distribution of boron (B) and carbon (C) throughout the hybrid material, excluding large-scale phase segregation.

**Figure 3:**
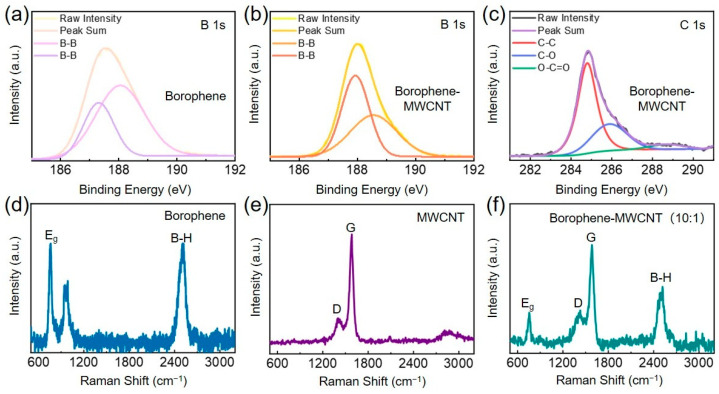
Spectroscopic Analysis of Borophene-MWCNT Heterostructures. (**a**) XPS spectrum of B 1s in borophene. (**b**) XPS spectrum of B 1s in the heterostructures. (**c**) XPS spectrum of C 1s in the heterostructures. (**d**) Raman spectrum of borophene. (**e**) Raman spectrum of MWCNT. (**f**) Raman spectrum of borophene-MWCNT heterostructures with a molar ratio of 10:1.

To elucidate the surface chemical composition and electronic structure of the as-prepared borophene and borophene-MWCNT heterostructures, X-ray photoelectron spectroscopy (XPS) analyses were systematically performed. The wide-scan XPS survey spectra (Figure S6) confirm the coexistence of boron and carbon species within the heterostructures, indicative of successful integration of borophene and MWCNT components. High-resolution XPS spectra of B 1s and C 1s regions were further deconvoluted to probe the nature of interfacial interactions. All binding energies were calibrated relative to the C 1s peak at 284.8 eV. The B 1s spectrum of pristine borophene displays two characteristic peaks at 187.3 eV and 188.0 eV (Figure 3a), corresponding to the presence of two distinct B-B bonding configurations intrinsic to the borophene lattice [12,16]. In the heterostructures, the B-B related peaks exhibit a pronounced blue shift to 187.9 eV and 188.5 eV (Figure 3b), suggesting a modified electronic environment of boron atoms, most likely arising from charge redistribution at the borophene-MWCNT interface. Simultaneously, the high-resolution C 1s spectrum of the heterostructures (Figure 3c) reveals multiple chemical states, including peaks at 284.8 eV (C-C), 285.9 eV (C-O), and 288.7 eV (O-C=O), reflecting the intrinsic surface chemistry of MWCNT and possible interfacial oxidation. The appearance and energy shift in these core-level features collectively indicate strong interfacial coupling between borophene and carbon nanotube. Notably, the observed chemical shifts point toward efficient interfacial electron transfer, further substantiating the formation of a chemically coherent heterojunction, critical for enhanced charge transport and sensor performance.

Raman spectroscopy was performed to interrogate the structural integrity and interfacial coupling within borophene-MWCNT heterostructures, probing phonon modes and electronic environments of constituent materials. Figure 3d–f present the Raman spectra of pristine borophene, MWCNT, and the as-fabricated borophene-MWCNT heterostructures, recorded at room temperature. Pristine borophene displays two characteristic vibrational modes centered at approximately 756 cm^−1^ (Eg mode) and 2504 cm^−1^ (B-H stretching mode), consistent with previously reported assignments [12]. In contrast, the Raman spectrum of MWCNT exhibits well-defined D and G bands at 1404 cm^−1^ and 1583 cm^−1^, respectively, when excited by a 325 nm laser, corresponding to disordered and graphitic carbon structures [23]. Raman spectrum of the heterostructures clearly integrates features from both components, exhibiting the B-H and Eg modes of borophene along with the D and G bands of MWCNT, thereby confirming their coexistence. Importantly, the B-H mode in the heterostructures exhibits a blue shift from 2504 cm^−1^ to 2519 cm^−1^, while the Eg mode remains at ~756 cm^−1^, suggesting subtle perturbations in the borophene lattice induced by interfacial interactions with MWCNT. Such spectral shifts are indicative of electronic coupling and potential charge transfer between the two phases, reinforcing the successful formation of an integrated borophene-MWCNT heterostructure with modified local bonding environments. These findings further corroborate the XPS results and validate the presence of strong interfacial synergy at the heterojunction level.

### 3.3. Humidity Sensing Using Borophene-MWCNT

**Figure 4:**
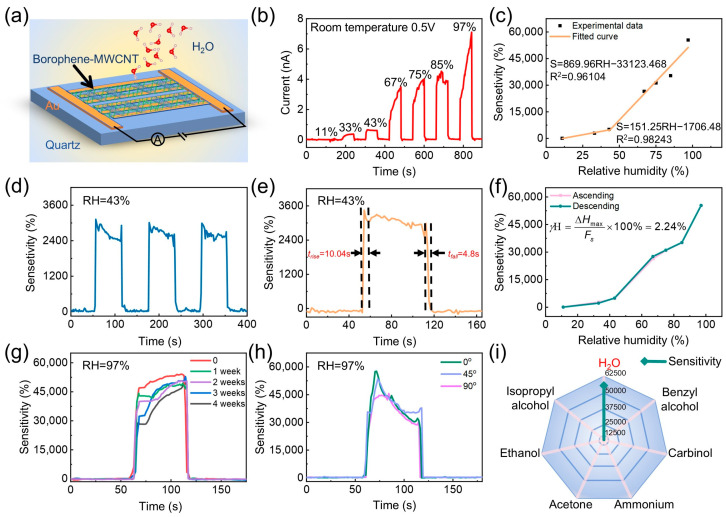
Fabrication and Characterization of the Borophene-MWCNT Humidity Sensor. (**a**) Schematic illustration of the sensor structure. (**b**) Real-time response of the sensor under varying relative humidity (RH) levels. (**c**) Fitted curve of sensor sensitivity as a function of RH. (**d**) Cycling stability of the sensor at 43% RH. (**e**) Response and recovery characteristics at 43% RH. (**f**) Hysteresis behavior of the sensor over the RH range of 11% to 97%. (**g**) Long-term operational stability at 97% RH. (**h**) Response curves under different bending angles: 0°, 45°, and 90°. (**i**) Sensitivity of the sensor toward different organic vapors.

To demonstrate the practical applicability of the borophene-MWCNT heterostructures in electronic sensing technologies, a resistive-type humidity sensor was fabricated, as schematically depicted in Figure 4a. The humidity-sensing performance was quantitatively assessed by recording the real-time current response of the device under systematically varied relative humidity (RH) conditions. A series of well-controlled humidity environments was established at room temperature using saturated salt solutions within hermetically sealed chambers. Specifically, RH levels of 11%, 33%, 43%, 67%, 75%, 85%, and 97% were achieved using lithium chloride (LiCl), magnesium chloride (MgCl_2_), potassium carbonate (K_2_CO_3_), copper (II) chloride (CuCl_2_), sodium chloride (NaCl), potassium chloride (KCl), and potassium sulfate (K_2_SO_4_), respectively. A near-zero RH baseline was generated using phosphorus pentoxide (P_2_O_5_) powder. The heterostructure-based sensor was exposed to each RH condition for a fixed duration of 60 s to ensure stabilization of the signal. The sensitivity (*S*) of the device was calculated using the standard expression:

(1) $$S(\%)=∆I/I_{0}\times 100\%=\left(I-I_{0}\right)/I_{0}\times 100\%$$

where I and $I_{0}$ denote the current measured at a given RH level and under dry air (0% RH), respectively.

As illustrated in Figure 4b and Figure S7, the current increased markedly from 12 pA at 0% RH to 7 nA at 97% RH, corresponding to an enhancement of over 550-fold. This pronounced current escalation underscores the excellent responsiveness of the borophene-MWCNT heterostructures to moisture stimuli. The fitted curve of sensor sensitivity under different relative humidity is plotted in Figure 4c. Under low RH conditions, the sensitivity exhibited a nearly linear correlation with RH, which can be fitted by the following relation:

(2) $$S=151.25RH-1706.48(R^{2}=0.98243)$$

In contrast, at higher RH levels, the sensitivity followed a steeper linear dependence, described by:

(3) $$S=869.96RH-33,123.468(R^{2}=0.96104)$$

These two-stage linear relationships reveal distinct sensing mechanisms at different humidity regimes, highlighting the heterostructure’s capacity for wide-range, high-resolution humidity detection. The strong correlation coefficients further indicate that RH values can be precisely inferred from the device response, offering a robust platform for intelligent environmental monitoring. As shown in Figure 4d, the borophene-MWCNT sensor exhibited a sensitivity of 3000% at 43% RH. Furthermore, the corresponding response and recovery times were 10.04 s and 4.8 s, respectively (Figure 4e), which are beneficial for real-time humidity monitoring. The repeatability of the borophene-MWCNT sensor was further assessed at various RH levels (Figure S8). The sensor’s repeatability was assessed across a wide range of relative humidity (RH) conditions. The borophene-MWCNT sensor retained robust response/recovery characteristics over consecutive measurement cycles, underscoring its cycling stability. Figure 4f shows the variation in sensor sensitivity during both forward and reverse scanning. The hysteresis ratio (*H*) is calculated using the following formula:

(4) $$H(\%)=\left(∆H_{max}\right)/F_{s}\times 100\%$$

where $∆H_{max}$ is defined as the maximum absolute difference between the output signals obtained during the forward and reverse scans at the same input value, and $F_{s}$ denotes the full-scale (maximum) output of the sensor. The calculated hysteresis ratio was found to be only 2.24%, indicating that the response depends solely on the real-time relative humidity, unaffected by the increasing or decreasing trend of RH.

To further quantify the sensor’s detection capability at trace humidity levels, the theoretical limit of detection (*LOD*) was calculated based on signal-to-noise analysis. First, the current was monitored in a stable low-humidity environment, and the root mean square value of the noise (*rms_noise_*) was computed for evaluation. Specifically, ten data points were extracted from the baseline section before the sensor was exposed to the humid environment (Figure 4b) and fitted (Figure S9). The *rms_noise_* was calculated using the following formula:

(5) $${rms}_{\mathrm{noise}}=\sqrt{\frac{\sum {\left(y_{i}-y\right)}^{2}}{N}}$$

where $y_{i}$ is the measured data point, y is the corresponding value calculated from the curve-fitting equation and N is the number of data points used in the curve fitting. The calculated value is approximately 0.0016 nA. Subsequently, the detection limit was determined using the following formula:

(6) $$LOD=\frac{3{rms}_{noise}}{K}$$

where K represents the slope of the linear fitting curve, with a value of 0.00158 nA/%RH. The calculation results demonstrate that the sensor exhibits a low *LOD* of 3.03% RH, further confirming its excellent ability to capture subtle moisture variations.

In addition to sensitivity and responsiveness, long-term operational stability is a critical parameter for practical sensing applications. Figure 4g displays the real-time dynamic response curves of the sensor exposed to 97% RH measured weekly over a month. It can be observed that the response waveform remains highly consistent, with only minor variations in amplitude. To strictly quantify this durability, the response amplitudes were normalized to the initial value (Week 0 defined as 100%), as presented in Figure S10. The calculation reveals that the borophene-MWCNT-based sensor maintained a high retention ratio of approximately 84.86% after four weeks of continuous environmental exposure, indicating excellent resistance to ambient degradation.

To elucidate the influence of composition on sensing performance, a series of borophene-MWCNT heterostructure-based humidity sensors were fabricated with varying molar ratios of borophene to MWCNT (5:1, 10:1, 30:1, and 50:1), alongside control samples based on pristine borophene and MWCNT. All sensors were prepared using an identical fabrication protocol to ensure comparability. As shown in Figure S11, each device exhibited stable and repeatable current responses under 97% RH, affirming the structural integrity and operational robustness of the sensor architecture. Remarkably, the heterostructures with a 10:1 borophene-to-MWCNT molar ratio demonstrated a superior sensing performance, achieving an extraordinary sensitivity of 55,000% at 97% RH-outperforming all other configurations by a significant margin. To evaluate device-to-device reproducibility, three independently fabricated borophene-MWCNT heterostructure humidity sensors with the same composition ratio (borophene:MWCNT = 10:1) were tested under a high-humidity environment of 97% RH (Figure S12). The extracted sensitivity values exhibit good consistency among the devices, yielding an average sensitivity of 55,184% with a sample standard deviation of 447%. This result can be expressed as 55,184% ± 447% (*n* = 3), indicating reliable fabrication reproducibility and stable sensing performance across different devices.

To contextualize these results within the broader field of humidity sensing, a comparative analysis was conducted against previously reported resistive-type humidity sensors based on various nanomaterials (Table S1). The sensitivity values reported in the literature are as follows: borophene-graphene (4200%) [19], carbon black (120%) [24], graphene (4.97%) [25], reduced graphene oxide (rGO, 36%) [26], rGO-MoS_2_ (23.85%) [27], black phosphorus (521%) [28], rGO-SnO_2_ (4600%) [29], MoS_2_ (2327%) [30], and borophene-BC_2_N quantum dots (22,001%) [31]. Notably, the borophene-MWCNT sensor developed in this study surpasses many existing counterparts, establishing a new benchmark for humidity sensor sensitivity among nanomaterial-based systems. These findings highlight the critical role of interfacial engineering and composition tuning in maximizing sensing performance, offering valuable insight for the rational design of next-generation humidity sensing platforms.

To further explore the practical applicability of borophene-MWCNT heterostructure sensors in flexible and wearable electronic systems, we integrated the sensor architecture onto thin, flexible polyethylene terephthalate (PET) substrates, replacing the conventional rigid glass platform. The mechanical adaptability of the device was systematically evaluated under three distinct deformation states: planar (flat), moderate bending (45°), and severe bending (90°) (Figure 4h). As illustrated in Figure 4h, the sensor consistently retained stable performance under all deformation conditions when exposed to 97% RH, exhibiting negligible fluctuations in response signal and sensitivity. These results demonstrate that the sensor can withstand significant mechanical deformation without compromising its functional integrity.

To rigorously evaluate the device’s selectivity, the sensor was exposed to several representative volatile organic compounds (VOCs) under testing conditions consistent with the humidity measurements. Specifically, the exposure time (60 s) and operating temperature were kept identical to ensure that adsorption equilibrium was reached for all analytes (Figure 4i and Figure S13). Gas sensing measurements were conducted in a sealed testing chamber equipped with an automated gas distribution system, enabling precise control over analyte concentrations. The sensor exhibited minimal current variation upon exposure to 100 ppm of common organic vapors, with sensitivities measured at 7.5% for ammonia, 3% for benzyl alcohol, 3.7% for acetone, 3.6% for carbinol, 2.5% for ethanol, and 11% for isopropyl alcohol. Notably, these responses are significantly lower than those observed for water vapor across the same concentration range, thereby confirming the sensor’s excellent selectivity toward humidity.

To elucidate the primary sensing mechanism and distinguish between electronic and ionic contributions, isotope substitution experiments were conducted using deuterium oxide (D_2_O) vapor (Figure S14). Sensor exhibits a rapid and robust current response to D_2_O (reaching ~700 pA), which is comparable in magnitude to that of H_2_O under identical conditions. The absence of a pronounced isotope effect indicates that ionic conduction is not the primary contributor to the sensing signal. Instead, this result confirms that the high sensitivity is dominantly governed by electronic transport modulation, providing a fundamental basis for understanding the sensing behavior.

An adsorption model was constructed based on first-principles density functional theory (DFT) calculations, as illustrated in Figure S15a. The adsorption energy (*E_ads_*) of the most stable configuration of the H_2_O molecule on the borophene-MWCNT heterostructures was calculated using the following equation:

(7) $$E_{ads}=E_{\mathrm{Borophene}-MWCNT-H_{2}O}-E_{H_{2}O}-E_{\mathrm{Borophene}-MWCNT}$$

where $E_{\mathrm{Borophene}-MWCNT-H_{2}O}$, $E_{H_{2}O}$ and $E_{\mathrm{Borophene}-MWCNT}$ represent the total energies of the borophene-MWCNT-H_2_O system, the H_2_O molecule, and the borophene-MWCNT heterostructures, respectively. The calculated adsorption energy is −0.16 eV, indicating a relatively strong interaction between the borophene-MWCNT heterostructures and the H_2_O molecule. To further visualize and quantify the charge transfer between the borophene-MWCNT and H_2_O molecules, charge density difference (CDD) analysis was performed. The CDD image was obtained using the equation:

(8) $$∆\rho =\rho_{\mathrm{Borophene}-MWCNT-H_{2}O}-\rho_{H_{2}O}-\rho_{\mathrm{Borophene}-MWCNT}$$

where $\rho_{\mathrm{Borophene}-MWCNT-H_{2}O}$, $\rho_{H_{2}O}$ and $\rho_{\mathrm{Borophene}-MWCNT}$ denote the charge densities of the adsorbed system, the isolated H_2_O molecule, and the pristine borophene-MWCNT heterostructures, respectively. The primary adsorption sites of the H and O atoms in the H_2_O molecule correspond to the yellow regions in Figure S15b, which indicate significant electron accumulation around the H_2_O molecule, thereby enhancing the electrical conductivity of the device. During this process, the Bader charge analysis result (Δq) is 0.014 e, suggesting that the H_2_O molecule gains electrons from MWCNT, corresponding to a p-type doping effect that increases the device’s conductivity.

The outstanding humidity sensing performance of the borophene-MWCNT heterostructures can be attributed to two synergistic mechanisms. First, the intimate interfacial contact between borophene and multiwalled carbon nanotubes facilitates reconstruction of the interfacial electronic structure and, under humidity stimulation, promotes interfacial charge redistribution, thereby enabling effective modulation of charge-carrier transport behavior, enhancing structural stability, and providing an active interfacial environment for the reversible adsorption of water molecules. Ultraviolet photoelectron spectroscopy (UPS) measurements reveal that the work functions of borophene and multi-walled carbon nanotubes (MWCNT) are 4.25 eV and 4.8 eV, respectively (Figure S16a,b). Combining the bandgap data of borophene determined from PL spectroscopy (Figure S17), we constructed the band structure of the heterostructures (Figure S16c). Because borophene has a lower work function, its Fermi level is higher than that of MWCNT, resulting in a shift in the Fermi level of borophene. To achieve Fermi level equilibrium, charge transfer occurs at the interface, and an interfacial barrier is formed. Upon water molecule adsorption, the interfacial charge redistribution and reduced barrier height lead to increased interfacial conduction, thereby enhancing the humidity sensing performance. Second, modulation of the Schottky barrier at the interface between the borophene-MWCNT heterostructures and the Au electrode further improves the sensing performance [19]. The band diagram in Figure S18 shows that the work function difference is consistent with the formation of a Schottky barrier at the interface, suggesting that this barrier can theoretically modulate carrier transport. When water molecules adsorb on the surface of the heterostructures, the Fermi level of borophene shifts toward the valence band [32,33], locally reducing the barrier height (Δ_SB_) and facilitating electron injection from the Au electrode. As the relative humidity increases, water molecule adsorption further promotes the formation of negatively charged H_2_O^δ−^ species, thereby increasing the sensor current [19]. This dynamic modulation of the Schottky barrier under humid conditions produces a pronounced current increase, amplifying the sensor response. Beyond previously reported humidity sensors, the borophene-MWCNT heterostructures offer mechanistic insight into humidity-induced Fermi-level equilibration and barrier-height modulation, providing a generalizable design principle for next-generation borophene-based hybrid devices.

In addition, the enhanced humidity-sensitive response of the sensor can also be attributed to the Grotthuss mechanism [34]. At low RH, only a sparse population of water molecules is adsorbed onto the surface of the sensing layer, where they partially dissociate into H_3_O^+^ and OH^−^ ions. The hydroxyl ions chemically bond with active sites on boron and carbon atoms, forming a chemisorbed layer that impedes charge transport, resulting in high resistance. As RH increases, additional water molecules are physisorbed onto the chemisorbed layer through hydrogen bonding, creating a primary physisorbed water layer. With further humidity elevation, multilayer adsorption occurs, leading to the formation of continuous water films across the sensing surface (Figure S19). These films provide a dynamic medium for rapid proton exchange via the Grotthuss mechanism (H_2_O + H_3_O^+^ → H_3_O^+^ + H_2_O) [35,36,37], drastically enhancing proton mobility and reducing the sensor’s electrical resistance. In contrast, the central finding of this study lies in two synergistic mechanisms within the borophene-MWCNT heterostructures, rather than the Grotthuss mechanism alone. The Grotthuss mechanism mainly plays an auxiliary role under high-humidity conditions. In summary, the sensing response of the borophene-MWCNT heterostructure is not governed by a single factor but arises from the synergistic interplay of multiple interfacial effects, collectively defined as the interfacial charge-transfer regulation mechanism. Specifically, this mechanism is driven by three key factors: first, the physisorption mechanism supported by the DFT-calculated adsorption energy of −0.16 eV, which is consistent with the observed reversible adsorption–desorption dynamics and suggests that it may contribute to the rapid response and recovery speeds. Second, the intimate interfacial contact between borophene and MWCNT, which facilitates the reconstruction of the interfacial electronic structure and Fermi-level alignment. Third, the humidity-responsive modulation of the Schottky barrier at the heterostructure/Au electrode interface, which effectively amplifies variations in charge-carrier transport. While multilayer water adsorption and the associated Grotthuss proton conduction process can provide an additional conductivity pathway under high relative humidity conditions, this process serves only as an auxiliary enhancement rather than playing a dominant role in determining the overall high-performance sensing behavior.

To assess the sensor’s reliability under realistic conditions, the influence of airflow and temperature was evaluated on two independently fabricated sensors (Figure S20). Under dry airflow at velocities of 5.0, 6.5, and 7.5 m/s, the electrical output remained highly stable, indicating negligible interference from aerodynamic disturbances (Figure S20a–f). Regarding temperature, a slight decrease in baseline current was observed over the range from 20 °C to 60 °C, suggesting that mechanisms such as enhanced carrier scattering may be at play. This thermal drift is orders of magnitude smaller than the humidity response signal (Figure S20g–i). These results confirm that the sensor output is dominantly governed by humidity, ensuring robust operation in practical wearable and ambient-sensing applications.

### 3.4. Application of Borophene-MWCNT Humidity Sensor

**Figure 5:**
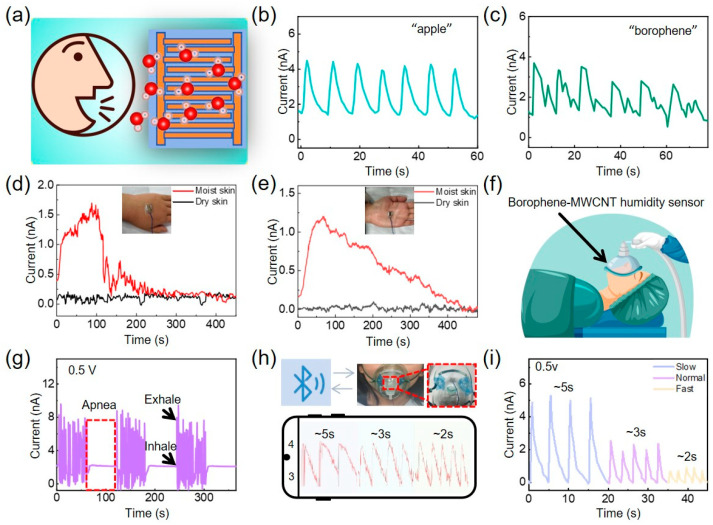
Human-Centric Applications of the Borophene-MWCNT Humidity Sensor. (**a**) Schematic diagram of the voice recognition system. (**b**,**c**) Cyclic sensor responses to spoken words: (**b**) “apple” and (**c**) “borophene”. (**d**) Real-time monitoring of skin moisture on the back of the hand. (**e**) Real-time monitoring of skin moisture on the palm. (**f**) Conceptual diagram of sensor applications in the medical field. (**g**) Sensor-based detection of breathing patterns, including apnea, exhalation, and inhalation. (**h**) Wireless monitoring of respiratory activity. (**i**) Sensor responses to varying respiration frequencies.

In contrast to conventional acoustic-based speech recognition technologies, the borophene-MWCNT humidity sensor offers unique advantages for assisting individuals with speech impairments or limited vocal capabilities, owing to humidity-resolved sensing mechanism. As shown in Figure 5b,c and Figure S21, the sensor is capable of capturing distinct current response signatures associated with different syllables when subjects silently mouthed words such as apple, borophene. Each articulation generated a reproducible and distinguishable signal pattern, arising from the subtle variations in exhaled breath humidity associated with different oral movements. This humidity-based sensing modality could allow for real-time monitoring of exhalation dynamics without reliance on sound waves, which could offer an alternative pathway for silent speech detection.

Skin moisture is a key physiological parameter intimately associated with both dermatological health and systemic well-being [30,38,39,40]. Given the human body’s lack of intrinsic humidity-specific sensory receptors, the use of electronic humidity sensors is indispensable for accurate assessment of cutaneous hydration levels [30]. To evaluate the practical applicability of the borophene-MWCNT sensor for skin moisture detection, flexible devices fabricated on PET substrates were conformally attached to the dorsum and palm surfaces of hands pre-rinsed with water, with adjacent untreated regions serving as internal controls (Figure 5d,e). Upon exposure to transient wetting, the sensors captured a hydration recovery process lasting approximately 3–7 min, during which the skin returned to its baseline moisture state. This observation underscores the sensor’s high sensitivity and responsiveness in tracking subtle variations in skin hydration in real time.

To demonstrate its practical applicability in wearable health monitoring platforms, the borophene-MWCNT humidity sensor was integrated into both medical-grade oxygen masks and conventional face masks (Figure 5f). As schematically shown in Figure S22, the system consists of four major components: a humidity sensor, a microcontroller (Arduino Nano), a Bluetooth communication module (HC-06), and a mobile host device. The sensor is connected to the microcontroller via a voltage divider circuit (R = 300 kΩ), enabling the analog signal to be read through the A0 port. The Bluetooth module communicates with the microcontroller via UART, and both components share a 5 V DC power supply with an operating current of 1 A. In the current prototype system, power is supplied through a USB port. On the software side, analog-to-digital conversion is employed for signal acquisition and serial communication, while a custom smartphone application receives and displays real-time breathing waveforms. This system achieves seamless hardware-software integration and stable wireless transmission under low power consumption.

Analog signals from port A0 reflect continuous voltage changes across the circuit. During exhalation, increased moisture content in the breath lowers the sensor’s resistance, causing a voltage rise across the protective resistor, manifested as current response peaks. Inhalation reduces ambient humidity, restoring the baseline signal (response valleys) [41]. Figure 5g showcases the device’s high temporal resolution in distinguishing distinct respiratory phases: apnea, exhalation, and inhalation. Of particular significance, the negligible signal variation observed during apnea episodes underscores the sensor’s potential utility for non-invasive sleep apnea detection. Real-time respiratory profiles, including diverse breathing patterns, were successfully visualized via a mobile application interface (Figure 5h and Video S1), demonstrating the feasibility of integrating the sensor with smart portable electronics. Furthermore, as shown in Figure 5i, the sensor reliably responds to different breathing rates corresponding to relaxed (~5 s), normal (~3 s), and accelerated (~2 s) respiratory states, highlighting its robustness and sensitivity across diverse physiological conditions.

## 4. Conclusions

In this study, we have successfully developed a high-performance humidity sensor based on a borophene-MWCNT heterostructure synthesized via chemical vapor deposition (CVD). By systematically optimizing the compositional ratio, the 10:1 molar ratio of borophene-to-MWCNT configuration was identified as the most effective, exhibiting exceptional sensing characteristics. Through interfacial charge-transfer engineering, the sensor achieved an ultra-high sensitivity of approximately 55,000% at 97% relative humidity (RH), representing 37-fold and 462-fold enhancements compared to sensors based solely on borophene and MWCNT, respectively. In addition to its outstanding sensitivity, the device demonstrated a broad detection range (11–97% RH), fast response/recovery times (10.04 s/4.8 s), excellent reproducibility, high selectivity, and long-term operational stability. Moreover, the sensor retained its performance under mechanical deformation, confirming its potential for integration into flexible and wearable electronics. The multifunctionality of the heterostructure-based sensor was preliminarily demonstrated through proof-of-concept applications in non-contact speech recognition, real-time skin hydration tracking, and wireless respiratory monitoring. These findings highlight the significant potential of borophene-based hybrid nanomaterials for advancing next-generation biomedical sensing platforms, particularly in the fields of personalized healthcare, intelligent diagnostics, and wearable medical technologies.

## Data Availability

Dataset available on request from the authors.

## Supplementary Materials

The following supporting information can be downloaded at: https://www.mdpi.com/article/10.3390/s26030976/s1, Figure S1: Growth curve of borophene-MWCNT heterostructures; Figure S2: SEM images of bare borophene, bare MWCNT and borophene-MWCNT heter- ostructures; Figure S3: AFM images and height profiles along the white lines (insets): (a) borophene, (b) MWCNT, and (c) borophene-MWCNT heterostructures; Figure S4: (a–c) High-resolution TEM images of the borophene-MWCNT interface captured at different regions, together with the corresponding selected-area electron diffraction (SAED) patterns; Figure S5: (a–d) STEM-HAADF-EDS elemental mapping images of the heterostructures; Figure S6: Full-range XPS survey spectra of borophene, multi-walled carbon nanotube (MWCNT), and borophene-MWCNT heterostructures with a molar ratio of 10:1; Figure S7: Real-Time Absolute Current Response Curves of the Borophene-MWCNT (10:1) Humidity Sensor; Figure S8: Cyclic response of the borophene-MWCNT (10:1) sensor at 11%, 75%, 85%, and 97% RH, respectively; Figure S9: Linear fit based on baseline data; Figure S10: Borophene-MWCNT humidity sensor long-term normalized stability; Figure S11: Response and recovery curves at 97% RH for sensors based on different materials; Figure S12: Dynamic response curves at 97% RH for three independently fabricated Borophene-MWCNT (10:1) heterostructure humidity sensors: (a) Sensor 1, 55,047%, (b) Sensor 2, 54,805%, (c) Sensor 3, 55,701%; Figure S13: Response curves of the heterostructured sensor upon exposure to typical organic vapors. Figure S14: Real-time dynamic response of the sensor to D_2_O vapor at 75% RH; Figure S15: DFT calculations of H_2_O adsorption on borophene-MWCNT heterostructures surface; Figure S16: UPS of borophene and MWCNT and the schematic band diagram of borophene-MWCNT heterostructures; Figure S17: PL spectrum of borophene; Figure S18: Band diagram of the contact between Au and borophene-MWCNT Schottky; Figure S19: Adsorption process of H_2_O on the surface of borophene-MWCNT heterostructures; Figure S20: Responses of the fabricated humidity sensors to environmental variations; Figure S21: Responses of the humidity sensor to spoken words; Figure S22: Schematic illustration of wireless monitoring system of respiratory behavior; Table S1: Comparison of reported resistive humidity sensors; Video S1: Wireless Sensing of Breathe Signals.

## Abbreviations

The following abbreviations are used in this manuscript:

CVD	Chemical Vapor Deposition
MBE	Molecular Beam Epitaxy
MWCNT	Multi-Walled Carbon Nanotube
SEM	Scanning Electron Microscopy
TEM	Transmission Electron Microscopy
AFM	Atomic Force Microscopy
HAADF-STEM	High-Angle Annular Dark-Field Scanning Transmission Electron Microscopy
XPS	X-ray Photoelectron Spectroscopy
UPS	Ultraviolet Photoelectron Spectroscopy
B	Boron
C	Carbon
RH	Relative Humidity
VOCs	Volatile Organic Compounds
