# Supplementary material for: Interfacial Charge-Transfer Engineering in Borophene–MWCNT Heterostructures for Multifunctional Humidity and Physiological Sensing
Source: Sensors (Basel). 2026 Feb 2;26(3):976. doi: 10.3390/s26030976 (PMC12899705; doi:10.3390/s26030976)
Supplement: Supplementary file 1 [file sensors-26-00976-s001.zip › Supplementary Information-sensors.pdf]

sensors

MDPI

Supplementary Information

Article

Interfacial charge-transfer engineering in borophene–MWCNT heterostructures for multifunctional humidity and physiological sensing

Anran Ma[1†], Tao Wang[1,2†], Zhilin Zhao[1†], Yi Liu[1], Maoping Xu[1], Shengxiang Gao[1], Rui Zhu[1], Jiamin Wu[1], Chuang Hou[3*], Guoan Tai[1*]

[1] State Key Laboratory of Mechanics and Control for Aerospace Structures, Laboratory of Intelligent Nano Materials, Devices of Ministry of Education, College of Aerospace Engineering, Nanjing University of Aeronautics and Astronautics, Nanjing 210016, China
[2] China Aerospace Science & Industry Academy of Information Technology，Beijing，100070，China
[3] Research Center for Advanced Science and Technology, The University of Tokyo, Tokyo,153-8505, Japan
* Correspondence: houchuang@g.ecc.u-tokyo.ac.jp; taiguoan@nuaa.edu.cn
† These authors contributed equally to this work.

This file includes:

Figure S1 Growth curve of borophene-MWCNT heterostructures.
Figure S2 SEM images of bare borophene, bare MWCNT and borophene-MWCNT heterostructures.
Figure S3 AFM images and height distribution histograms along the white arrows (insets): (a) borophene, (b) MWCNT, and (c) borophene-MWCNT heterostructures.
Figure S4 (a-c) High-resolution TEM images of the borophene-MWCNT interface captured at different regions, together with the corresponding selected-area electron diffraction (SAED) patterns.
Figure S5 (a-d) STEM-HAADF-EDS elemental mapping images of the heterostructures.
Figure S6 Full-range XPS survey spectra of borophene, multi-walled carbon nanotube (MWCNT), and borophene-MWCNT heterostructures with a molar ratio of 10:1.
Figure S7 Real-Time Absolute Current Response Curves of the Borophene-MWCNT (10:1) Humidity Sensor.
Figure S8 Cyclic response of the borophene-MWCNT (10:1) sensor at 11%, 75%, 85%, and 97% RH, respectively.
Figure S9 Linear fit based on baseline data.
Figure S10 Borophene-MWCNT humidity sensor long-term normalized stability.
Figure S11 Response and recovery curves at 97% RH for sensors based on different materials.
Figure S12 Dynamic response curves at 97 % RH for three independently fabricated Borophene-MWCNT (10:1) heterostructure humidity sensors: (a) Sensor 1, 55,047 %, (b) Sensor 2, 54,805 %, (c) Sensor 3, 55,701 %.
Figure S13 Response curves of the heterostructured sensor upon exposure to typical organic vapors.
Figure S14 Real-time dynamic response of the sensor to D_2O vapor at 75% RH.

Figure S15 DFT calculations of H_2O adsorption on borophene-MWCNT heterostructures surface.
Figure S16 UPS of borophene and MWCNT and the schematic band diagram of borophene-MWCNT heterostructures.
Figure S17 PL spectrum of borophene.
Figure S18 Band diagram of the contact between Au and borophene-MWCNT Schottky.
Figure S19 Adsorption process of H_2O on the surface of borophene-MWCNT heterostructures.
Figure S20 Responses of the fabricated humidity sensors to environmental variations.
Figure S21 Responses of the humidity sensor to spoken words.
Figure S22 Schematic illustration of wireless monitoring system of respiratory behavior.
Table S1 Comparison of reported resistive humidity sensors.

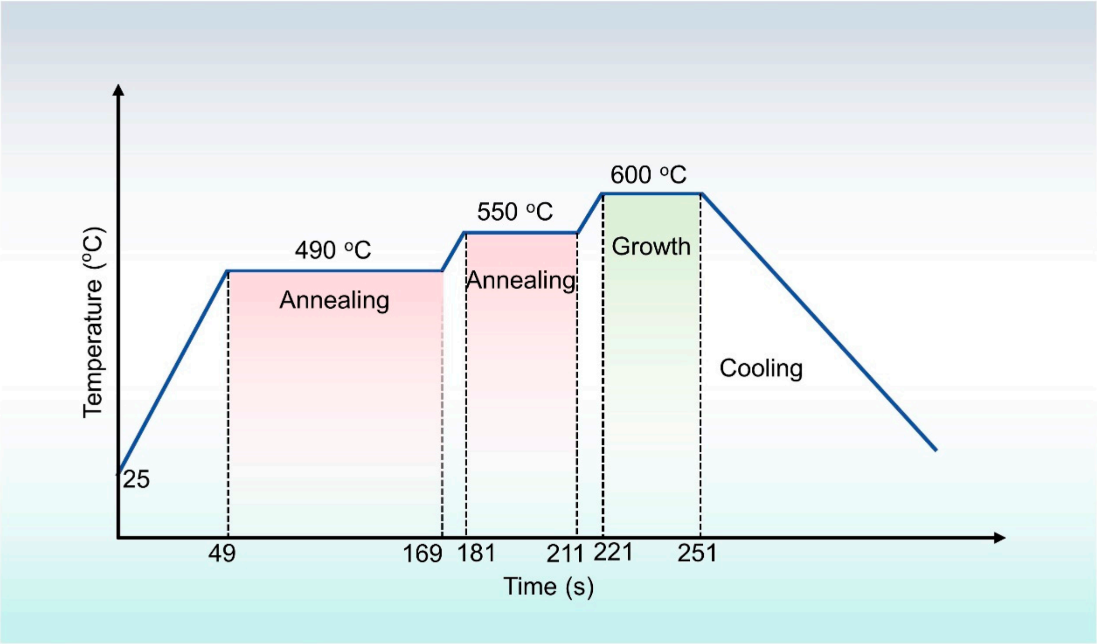

Figure S1 Growth curve of borophene-MWCNT heterostructures.

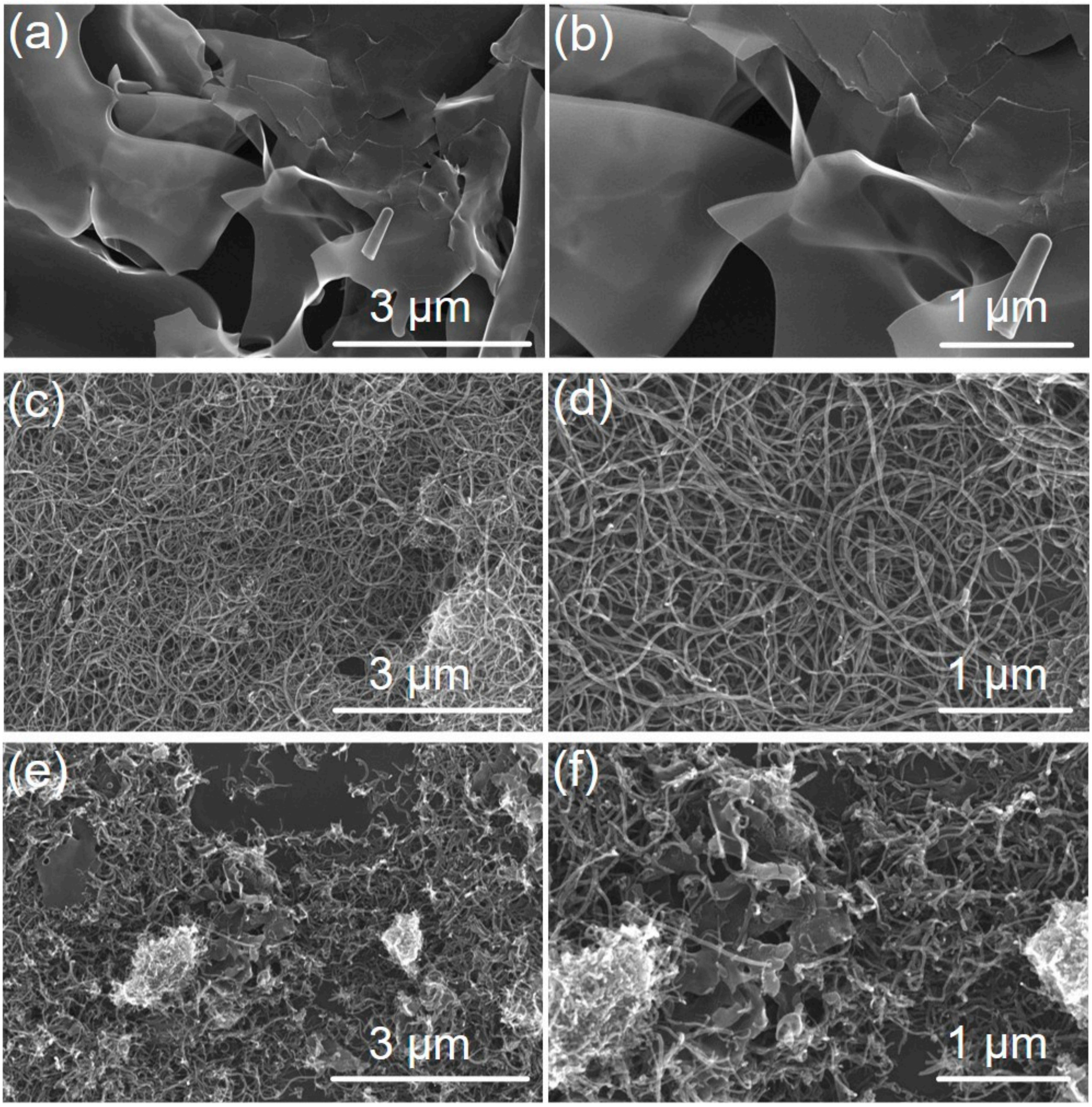

Figure S2 SEM images of (**a**, **b**) bare borophene, (**c**, **d**) bare MWCNT and (**e**, **f**) borophene-MWCNT heterostructures.

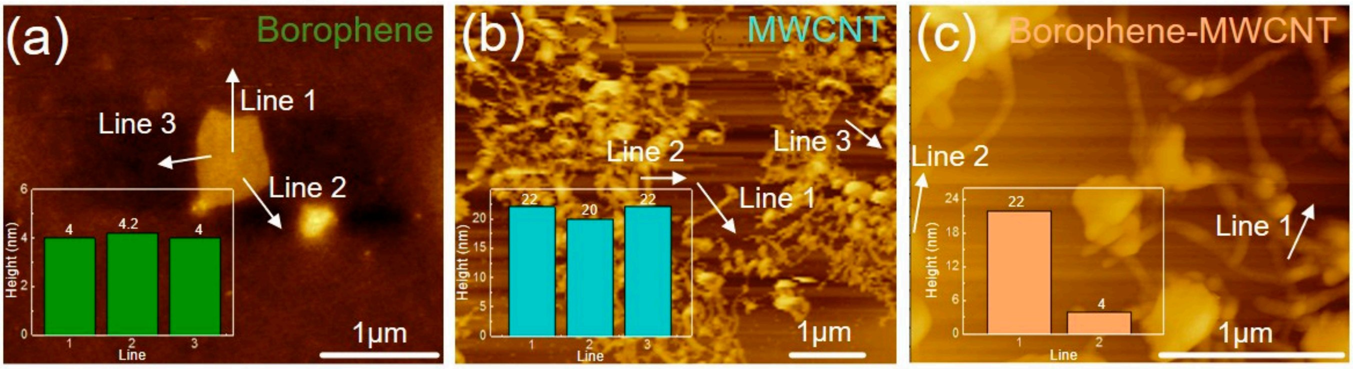

Figure S3 AFM images and height profiles along the white dashed lines (insets): (a) borophene, (b) MWCNT, and (c) borophene-MWCNT heterostructures.

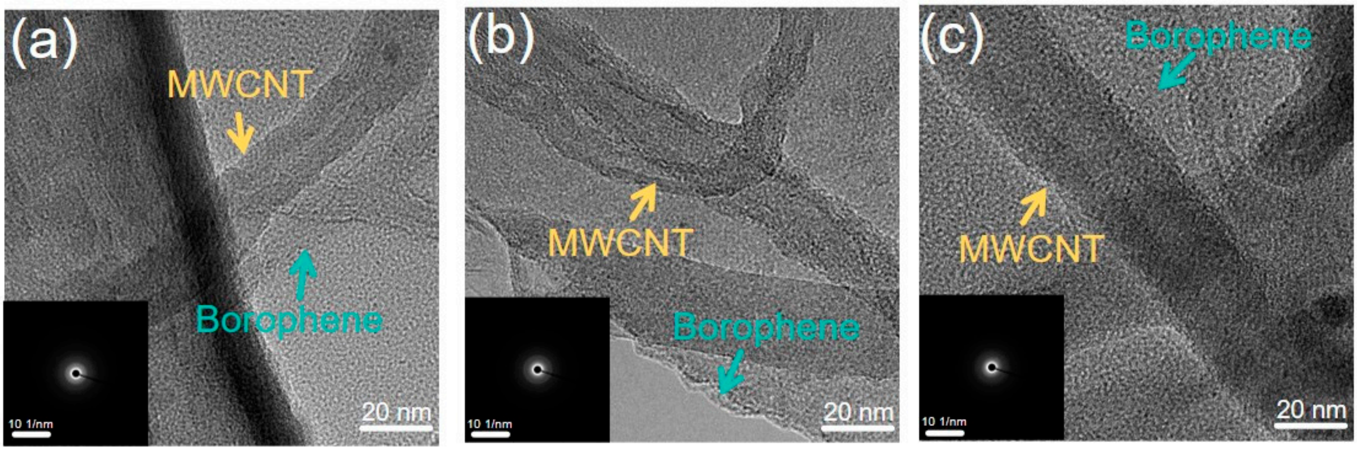

Figure S4 (**a-c**) High-resolution TEM images of the borophene-MWCNT interface captured at different regions, together with the corresponding selected-area electron diffraction (SAED) patterns.

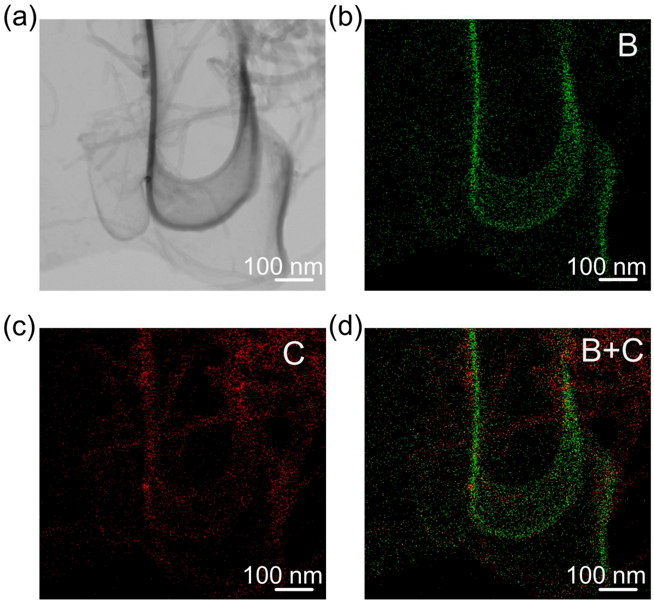

Figure S5 (**a**-**d**) STEM-HAADF-EDS elemental mapping images of the heterostructures.

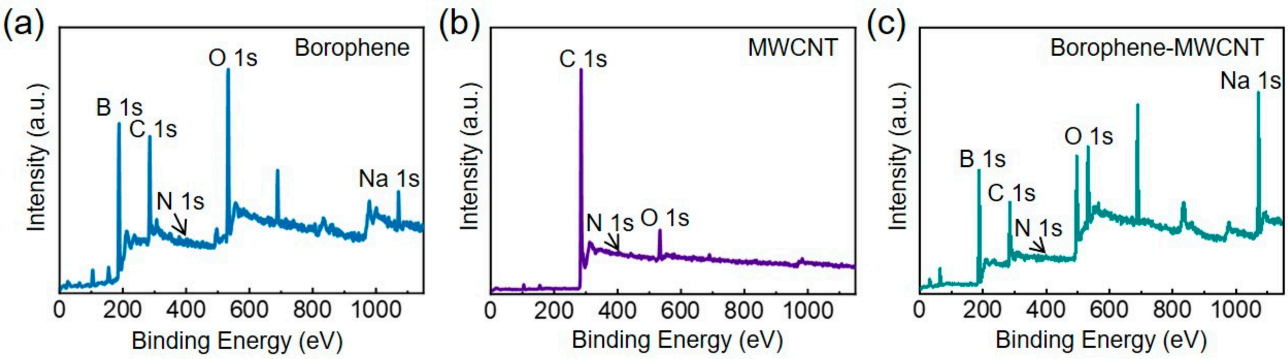

Figure S6 Full-range XPS survey spectra of borophene, multi-walled carbon nanotube (MWCNT), and borophene-MWCNT heterostructures with a molar ratio of 10:1.

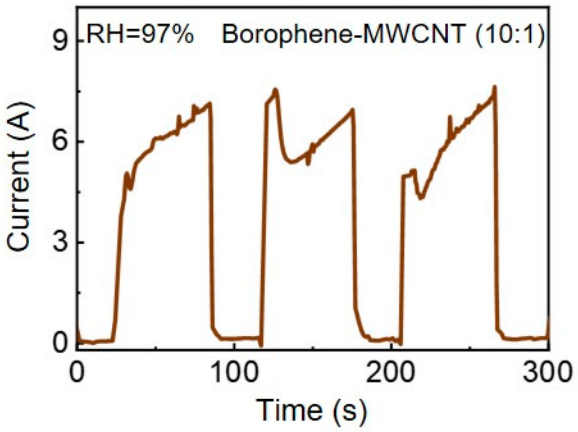

Figure S7 Real-Time Absolute Current Response Curves of the Borophene-MWCNT (10:1) Humidity Sensor.

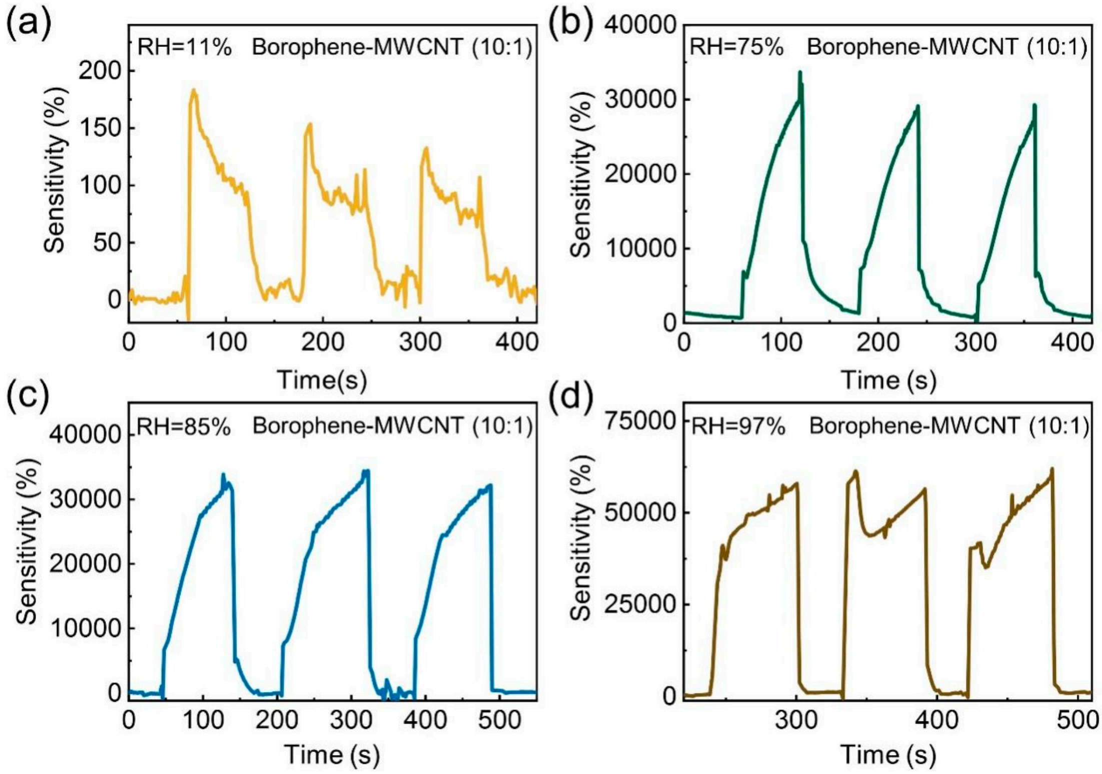

Figure S8 Cyclic response of the borophene-MWCNT (10:1) sensor at 11%, 75%, 85%, and 97% RH, respectively.

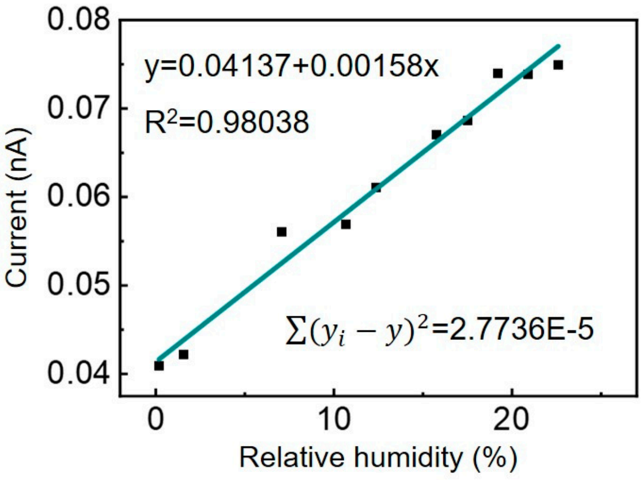

Figure S9 Linear fit based on baseline data.

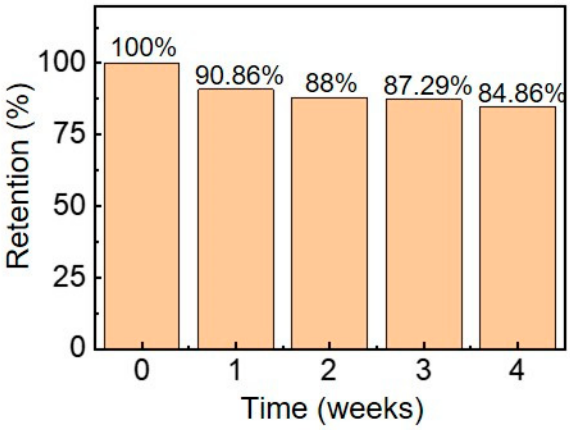

Figure S10 Borophene-MWCNT humidity sensor long-term normalized stability.

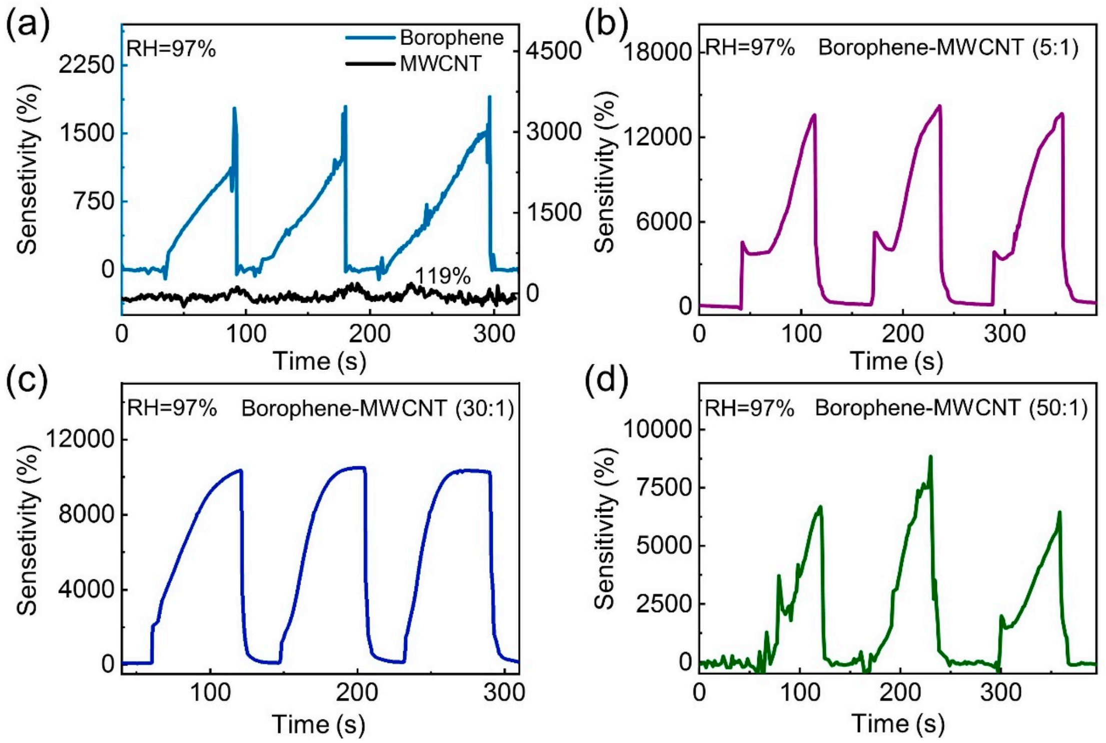

Figure S11 Response and recovery curves at 97% RH for sensors based on different materials: (**a**) borophene and MWCNT, (**b**) borophene-MWCNT (5:1), (**c**) borophene-MWCNT (30:1), and (**d**) borophene-MWCNT (50:1).

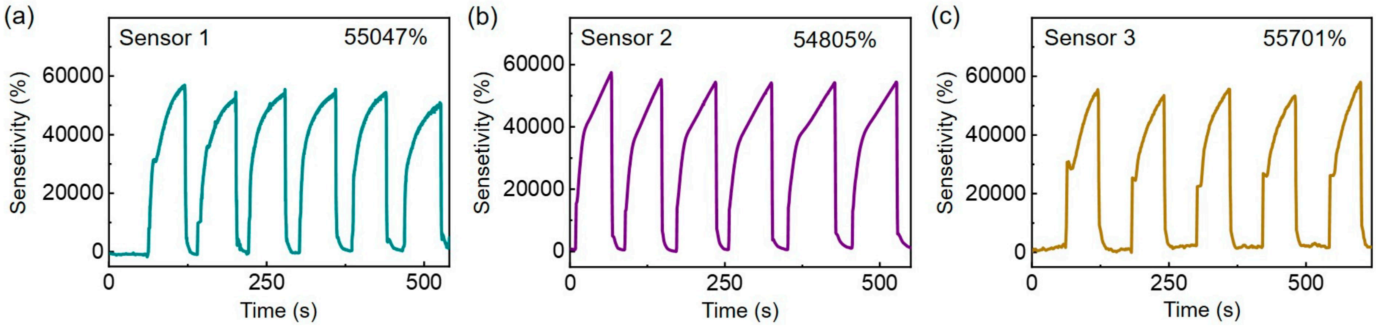

Figure S12 Dynamic response curves at 97 % RH for three independently fabricated Borophene-MWCNT (10:1) heterostructure humidity sensors: (a) Sensor 1, 55,047 %, (b) Sensor 2, 54,805 %, (c) Sensor 3, 55,701 %.

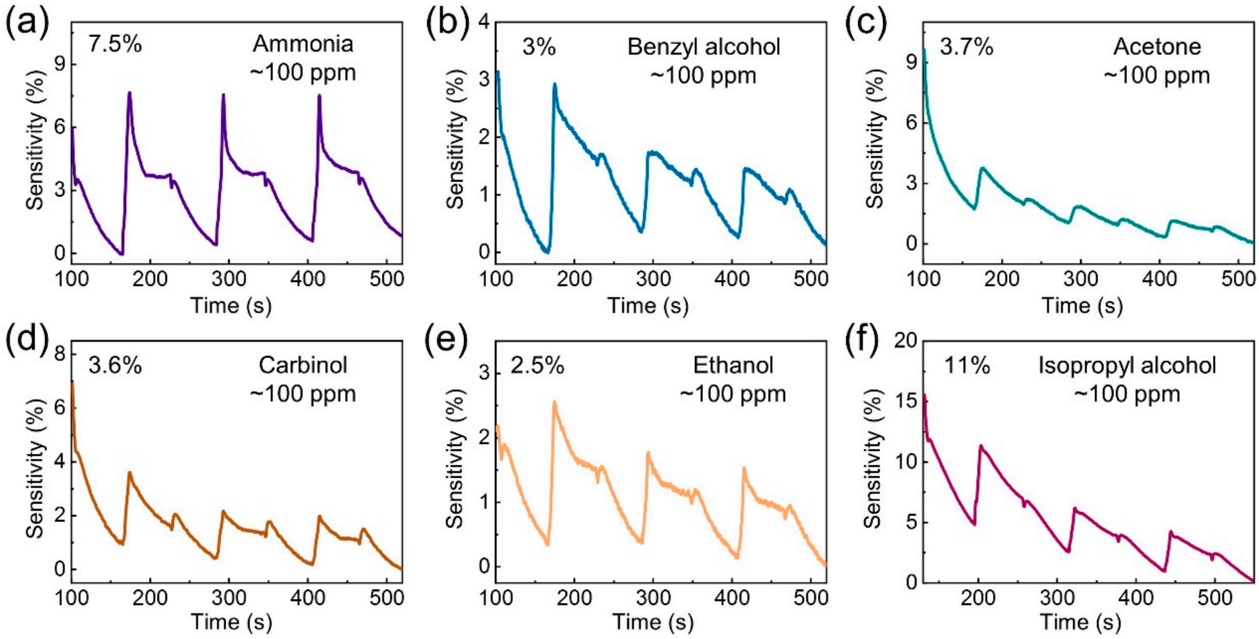

Figure S13 Response curves of the heterostructured sensor upon exposure to typical organic vapors: (**a**) ammonia, (**b**) benzyl alcohol, (**c**) acetone, (**d**) carbinol, (**e**) ethanol, and (**f**) isopropyl alcohol.

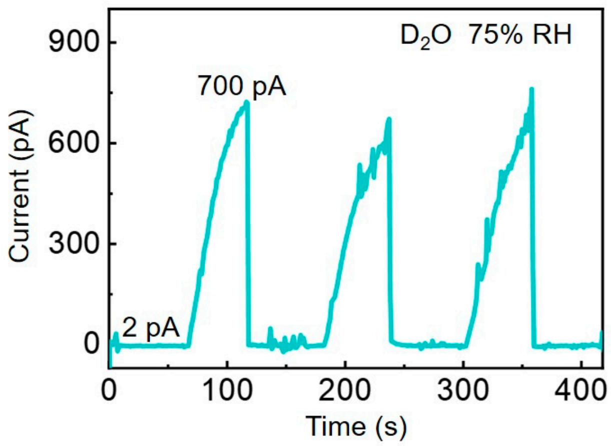

Figure S14 Real-time dynamic response of the sensor to D_2O vapor at 75% RH.

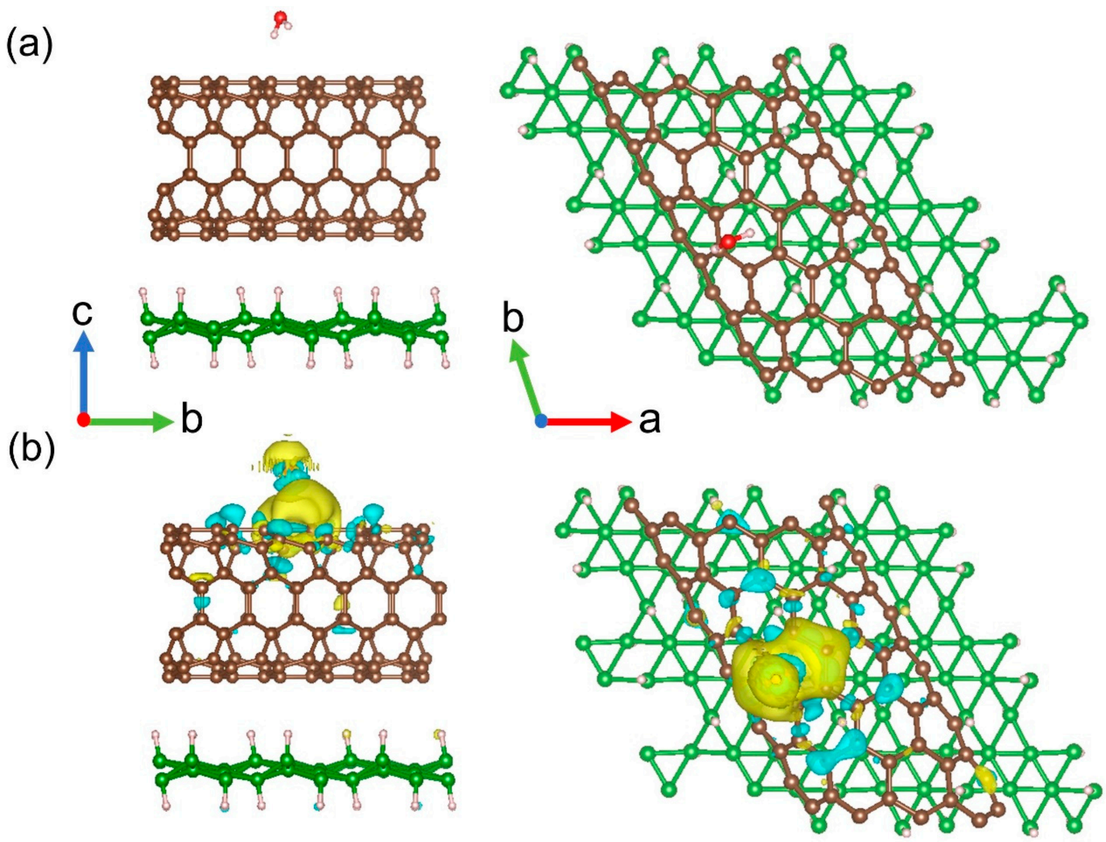

Figure S15 DFT calculations of H_2O adsorption on borophene-MWCNT heterostructures surface. (**a**) Side and top views of the borophene-MWCNT heterostructures with the most stable configuration. (**b**) Charge density difference (CDD) plot for H_2O adsorption on the borophene-MWCNT heterostructures.

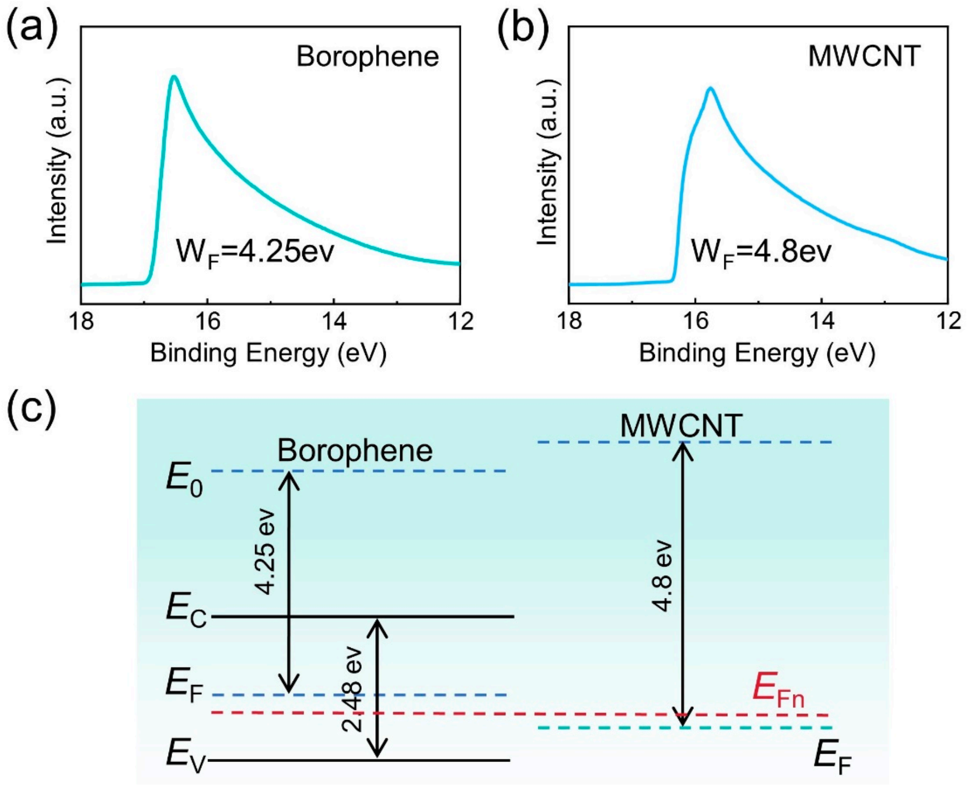

Figure S16 (**a**) UPS of borophene. (**b**) UPS of MWCNT. (**c**) Band diagram of borophene -MWCNT heterostructures.

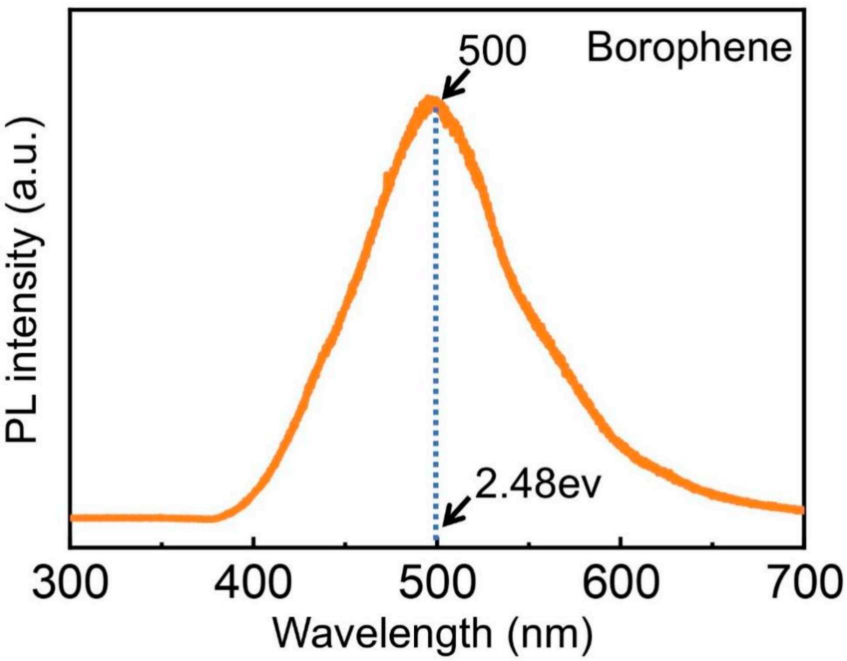

Figure S17 PL spectrum of borophene.

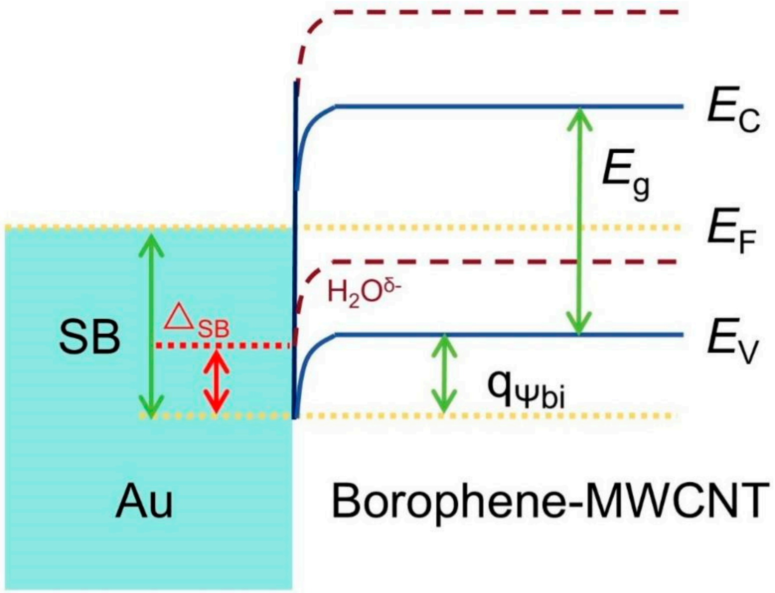

Figure S18 Band diagram of the contact between Au and borophene-MWCNT Schottky.

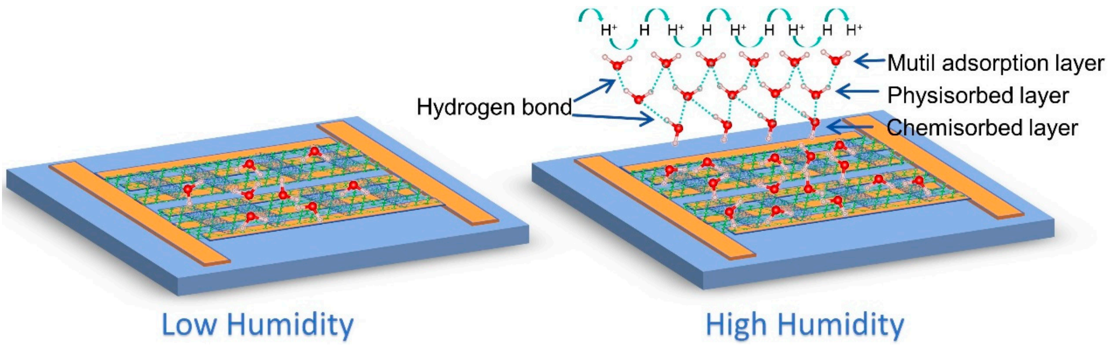

Figure S19 Adsorption process of H_2O on the surface of borophene-MWCNT heterostructures.

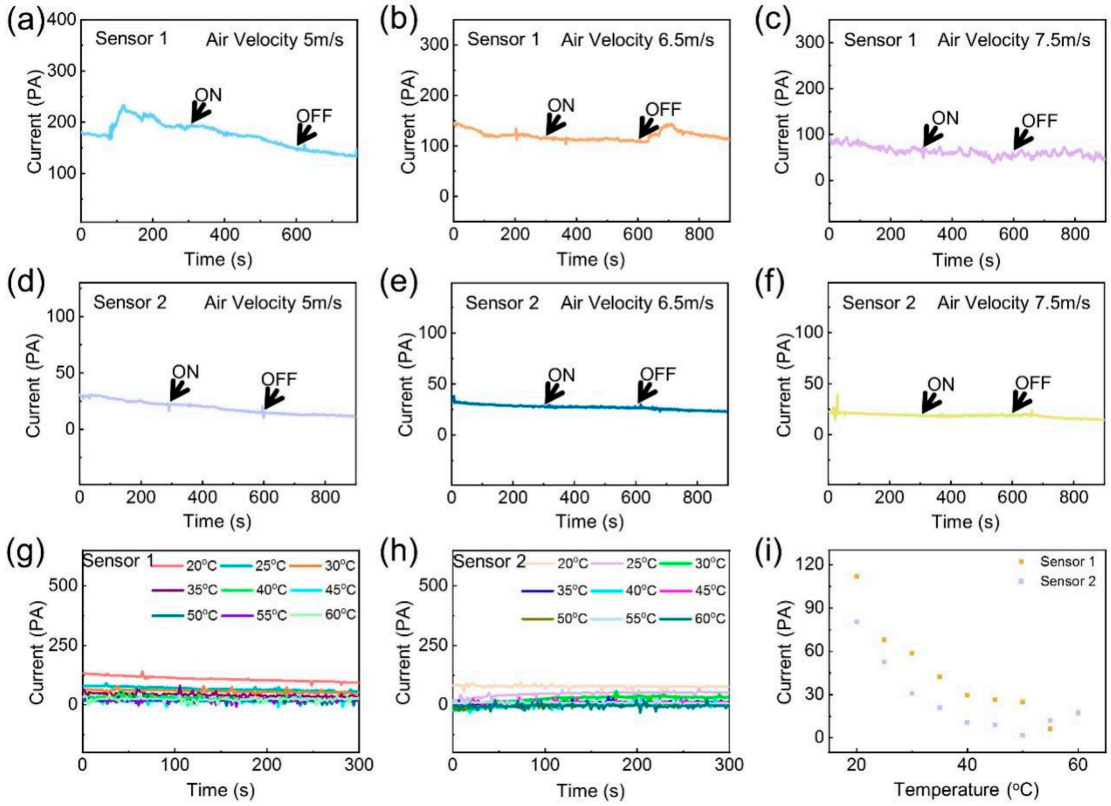

Figure S20 Responses of the fabricated humidity sensors to environmental variations: (**a**-**f**) wind and (**g**-**h**) temperature. (**i**) Correlation between sensor temperature and output current.

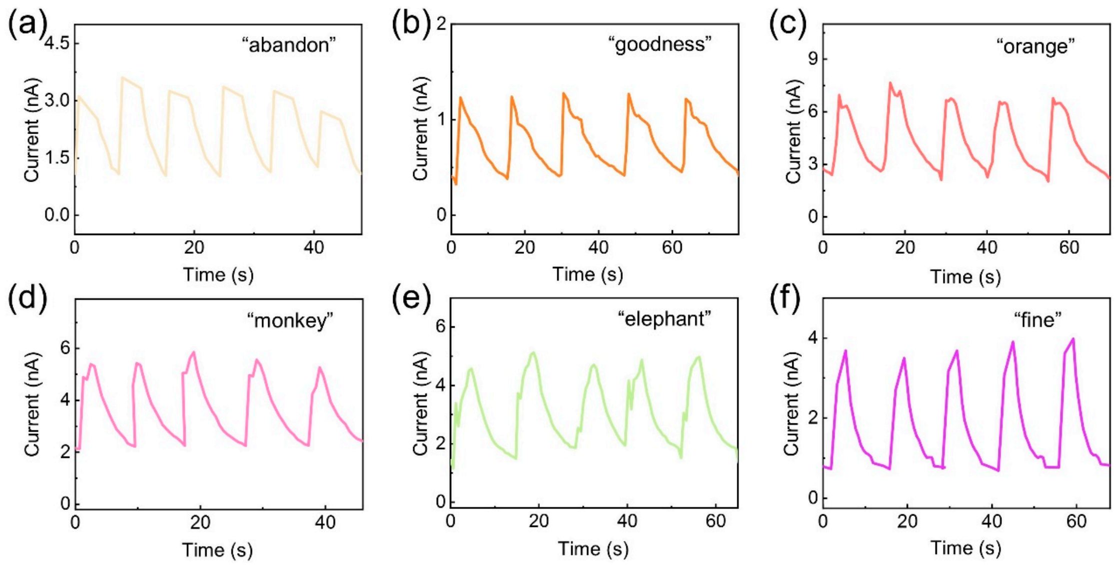

Figure S21 Responses of the humidity sensor to spoken words: (**a**) 'abandon', (**b**) 'goodness', (**c**) 'orange', (**d**) 'monkey', (**e**) 'elephant', and (**f**) 'fine'.

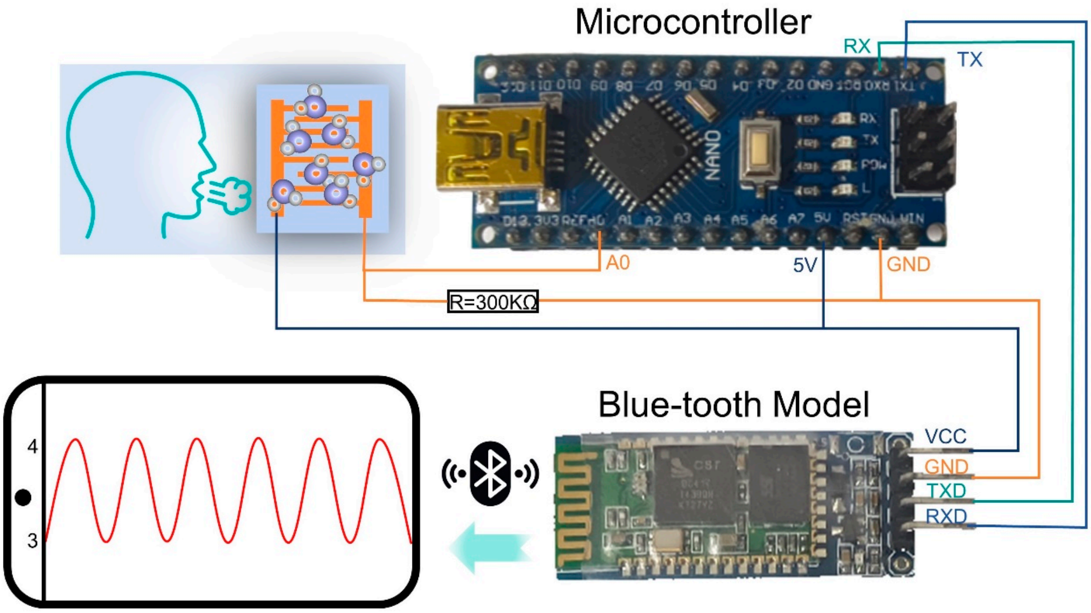

Figure S22 Schematic illustration of wireless monitoring system of respiratory behavior.

Table S1 Comparison of reported resistive humidity sensors

Material	Sensitivity (%)	Measured RH value	Humidity range	Response(s) /recovery (s)	Reference
Borophene-graphene	4200	85%	11-85%	10.5/8.3	19
Carbon black	120	90%	30-90%	10/6	24
Graphene	4.97	97%	12-97%	31/72	25
RGO	36	97%	11-97%	108/94	26
RGO/MoS_2	23.85	90%	10-90%	30/253	27
Black phosphorus (BP)	521	97%	11–97%	101/26	28
RGO-SnO_2	4600	95%	11-95%	10/60	29
MoS_2	2327	89.5%	17.2-89.5%	140/80	30
Borophene-BC_2N	22001	97%	11-97%	11.82/1.41	31
**Borophene-MWCNT**	**55000**	**97%**	**11-97%**	**10.04/4.8**	**This work**
